# Vasomotor function in rat arteries after ex vivo and intragastric exposure to food-grade titanium dioxide and vegetable carbon particles

**DOI:** 10.1186/s12989-018-0248-2

**Published:** 2018-02-26

**Authors:** Ditte Marie Jensen, Daniel Vest Christophersen, Majid Sheykhzade, Gry Freja Skovsted, Jens Lykkesfeldt, Rasmus Münter, Martin Roursgaard, Steffen Loft, Peter Møller

**Affiliations:** 1 Department of Public Health, Section of Environmental Health, Faculty of Health and Medical Sciences, University of Copenhagen, Øster Farimagsgade 5A, Building 5B, 2nd Floor, DK-1014 Copenhagen K, Denmark; 2 Department of Drug Design and Pharmacology, Section of Molecular and Cellular Pharmacology, Faculty of Health and Medical Sciences, University of Copenhagen, Universitetsparken 2, 22, 2100 Copenhagen, Denmark; 3 Experimental Animal Models, Department of Veterinary and Animal Sciences, Faculty of Health and Medical Sciences, University of Copenhagen, Ridebanevej 9, DK-1870 Frederiksberg C, Denmark; 4 Colloids and Biological Interfaces, Department of Micro- and Nanotechnology, Technical University of Denmark, 2800 Kongens Lyngby, Denmark

**Keywords:** Vasomotor function, E153, E171, Vegetable carbon, Titanium dioxide, Nanoparticles, Oxidative stress, Gastrointestinal exposure, Endothelial dysfunction

## Abstract

**Background:**

Humans are continuously exposed to particles in the gastrointestinal tract. Exposure may occur directly through ingestion of particles via food or indirectly by removal of inhaled material from the airways by the mucociliary clearance system. We examined the effects of food-grade particle exposure on vasomotor function and systemic oxidative stress in an ex vivo study and intragastrically exposed rats.

**Methods:**

In an ex vivo study, aorta rings from naïve Sprague-Dawley rats were exposed for 30 min to food-grade TiO_2_ (E171), benchmark TiO_2_ (Aeroxide P25), food-grade vegetable carbon (E153) or benchmark carbon black (Printex 90). Subsequently, the vasomotor function was assessed in wire myographs. In an in vivo study, lean Zucker rats were exposed intragastrically once a week for 10 weeks to vehicle, E171 or E153. Doses were comparable to human daily intake. Vasomotor function in the coronary arteries and aorta was assessed using wire myographs. Tetrahydrobiopterin, ascorbate, malondialdehyde and asymmetric dimethylarginine were measured in blood as markers of oxidative stress and vascular function.

**Results:**

Direct exposure of E171 to aorta rings ex vivo increased the acetylcholine-induced vasorelaxation and 5-hydroxytryptamine-induced vasocontraction. E153 only increased acetylcholine-induced vasorelaxation, and Printex 90 increased the 5-hydroxytryptamine-induced vasocontraction, whereas Aeroxide P25 did not affect the vasomotor function. In vivo exposure showed similar results as ex vivo exposure; increased acetylcholine-induced vasorelaxation in coronary artery segments of E153 and E171 exposed rats, whereas E171 exposure altered 5-hydroxytryptamine-induced vasocontraction in distal coronary artery segments. Plasma levels of markers of oxidative stress and vascular function showed no differences between groups.

**Conclusion:**

Gastrointestinal tract exposure to E171 and E153 was associated with modest albeit statistically significant alterations in the vasocontraction and vasorelaxation responses. Direct particle exposure to aorta rings elicited a similar type of response. The vasomotor responses were not related to biomarkers of systemic oxidative stress.

## Background

Humans are continuously exposed to particles through the gastrointestinal (GI) tract. Particles are widely used in food products and drugs as pigments or as additives. Another important source of oral exposure is inhaled particles because particles in the upper respiratory tract are removed by the mucociliary clearance system and subsequently swallowed [1]. It has been estimated that 60% of the inhaled mass of particles is cleared to the stomach after 200 h [2]. Thus, inhalation of pigments such as TiO_2_ inadvertently gives rise to GI exposure. Although there is an increase in the use of engineered nanoparticles as food additives, there is limited knowledge about the possible detrimental effects of oral exposure.

There is a relatively large body of literature showing that pulmonary exposure to particles is associated with vasomotor dysfunction and progression of atherosclerosis [3, 4], which are important intermediate steps in the development of cardiovascular disease. The underlying pathophysiological mechanisms are complex and poorly understood; however particle-generated oxidative stress seems to be a key component [5]. We have previously shown that oral exposure to nanosized carbon black in the form of Printex 90 was associated with endothelial dysfunction, which was observed as reduced acetylcholine (ACh)-induced vasorelaxation in the aorta, in both lean and obese Zucker rats [6]. Endothelial dysfunction, a hallmark in development of atherosclerosis, is characterized by endothelial nitric oxide synthase (eNOS) uncoupling and a concomitant shift from production of nitric oxide (NO) to superoxide anion radicals and peroxynitrite. This event results in decreased NO bioavailability, which is associated with vasomotor dysfunction. The increased production of superoxide anion radicals and peroxynitrite promotes a local milieu of oxidative stress in the endothelial cells. Uncoupling of eNOS can be caused by reduced bioavailability of tetrahydrobiopterin (BH_4_), which is a cofactor for eNOS [7]. BH_4_ reacts readily with reactive oxygen species and is thus susceptible to depletion during oxidative stress [7]. It has been shown that ascorbic acid improves the NO-dependent vasorelaxation in arteries via higher BH_4_ bioavailability [8], further emphasizing the role of oxidative stress involved in vasomotor dysfunction. Systemic oxidative stress is also considered to be an important intermediate step in the development of vasomotor dysfunction. Decreased levels of antioxidants (e.g., ascorbic acid) or increased levels of lipid oxidation products such as malondialdehyde (MDA) are typically used as indicators of oxidative stress.

This study aimed to investigate the effect of repeated intragastric administration of food-grade particles on vasomotor function and systemic oxidative stress. Due to the analogy between air pollution particles and nanomaterials, we hypothesize that intragastric exposure to particles could result in similar effects as inhalation exposure. Carbon-based materials such as Printex 90 and other nanomaterials are not likely to be ingested on a regular basis by consumers. However, the European Food and Safety Authority (EFSA) has approved the use of carbon-based material from vegetable origin (E153) and TiO_2_ (E171) as food coloring substances [9, 10] and there is no established upper threshold limit of intake. E153 is used as a black colorant in candy and as a pharmaceutical product for the treatment of acute poisoning and diarrhea. E171 is used as a white colorant in e.g., candy and dressings. It should be emphasized that these coloring agents are not nanomaterials per se, although there is a fraction of particles in the nanosize range in the samples. Nevertheless, they are highly relevant materials in particle toxicology because humans are exposed to these particles on a daily basis. First, we compared the effect of E171 and E153 to benchmark counterparts (Aeroxide P25 and Printex 90, respectively) on vasomotor function ex vivo in isolated aorta segments from naïve rats. Aeroxide P25 and Printex 90 have been used extensively in nanotoxicology and inhalation toxicology. We have used Aeroxide P25 and Printex 90 as benchmark particles, but there is not sufficient experimental evidence to regard them as positive controls for cardiovascular disease endpoints. Subsequently, we investigated in vivo effects, i.e., vasomotor function in the aorta and coronary arteries and systemic oxidative stress in lean Zucker rats after repeated oral administration of E171 and E153. The lean Zucker rat was chosen as an animal model, because of previous findings of endothelial dysfunction in the aorta in animals exposed to Printex 90 [6].

We have used one weekly exposure because repeated oral gavages may cause adverse effects in the rats. In addition, there is day-to-day variation in the exposure to E153 and E171, related to the intake of food (i.e. high exposure on certain days and little exposure on other days of the week). According to EFSA, the average daily intake of E171 is 1.8–10.4 mg/kg in children, whereas the 95% percentile is 4.9–32.4 mg/kg per day [10]. This corresponds to average weekly accumulated doses of 34 to 227 mg/kg for high-consumers. In comparison, the intake of E171 is lower in the elderly (mean: 0.4–4.5 mg/kg/day; 95% percentile: 1.2–10.7 mg/kg/day). The vegetable carbon doses were selected from an earlier study with Printex 90 [6]. It has been estimated that the mean dietary exposure to vegetable carbon is 3.0–29.7 and 3.7 mg/kg bw per day in children and adults respectively [9]. The high-level consumers have a daily intake of 15.3–79.1 and 28.1 mg/kg bw in children and adults, respectively [9]. Thus, the doses of E153 (i.e., 0.64 and 6.4 mg/kg) corresponds to the average daily dose in humans. The purpose of the ex vivo study was to compare the effect of the food-grade particles with their respective pigment-grade particles. Thus, the concentrations are rather high as compared to realistic concentrations after intake of E153 and E171. The concentrations of 10 and 100 μg/ml of E153 were identical to previous experiments on Printex 90 in aorta rings from mice [11]. The concentrations of TiO_2_ in the ex vivo study was based on the average daily intake in children (i.e. 10.4 mg/kg/day), bodyweight (35 kg), blood volume (2500 ml) and either complete or 10% uptake of particles.

## Results

### Particle characterization

**Fig. 1:**
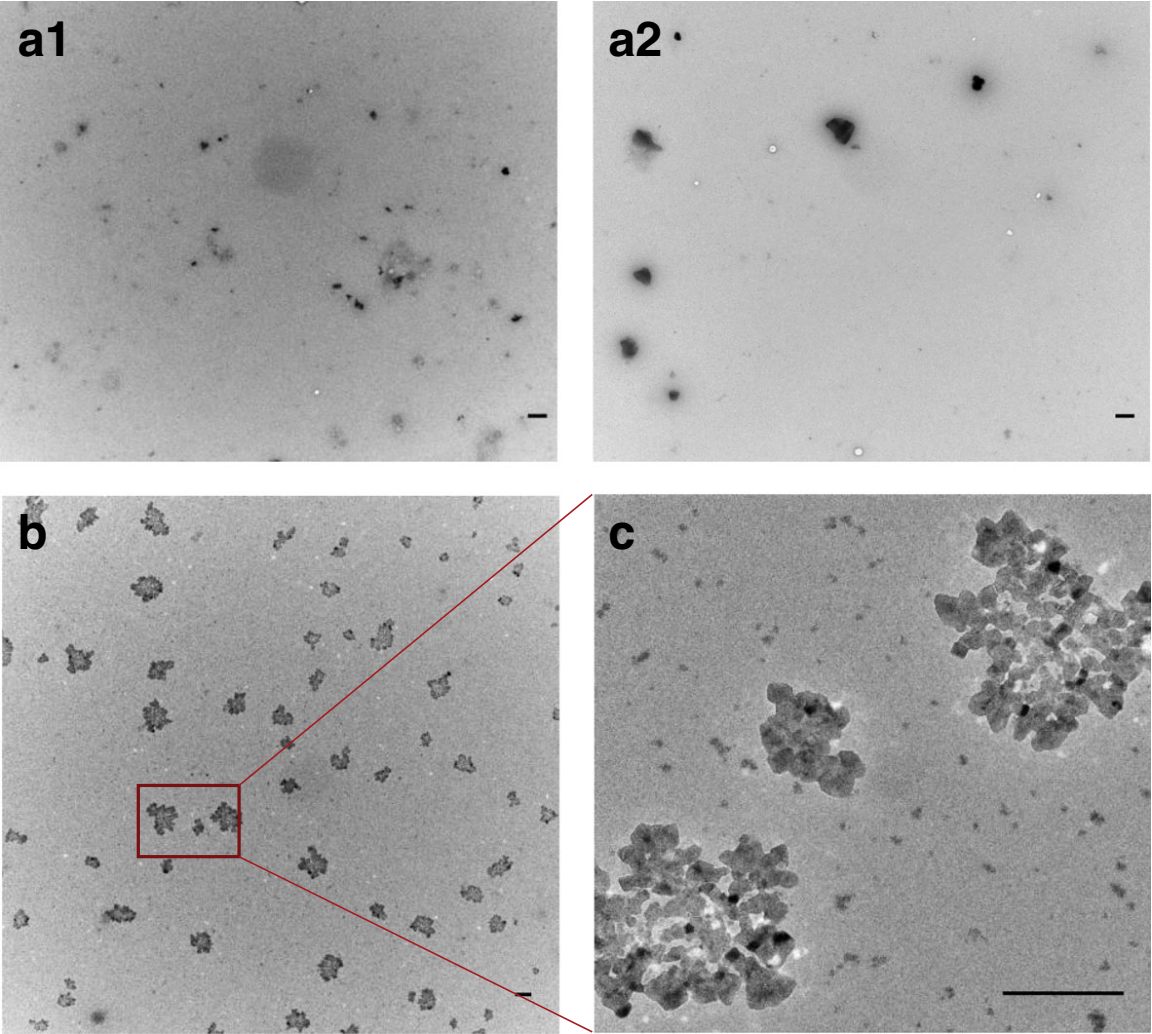
TEM images of E153 and E171. Dry particle size and morphology of E153 and E171 was determined by TEM images. (**a1**) and (**a2**) are images of E171, magnification 5800×. Three size groups of E171 particles were observed in the pictures. (**b**) and (**c**) are images of E153, magnification 5800× (**b**) and 44,000× (**c**). Two size groups of particles were observed for E153. Scale bars are 500 nm

The particle size was assessed in dry form by transmission electron microscopy (TEM) (Fig. 1 and Table 1). Three size groups for E171 were observed from the TEM images; 135 ± 46 nm, 305 ± 61, 900 ± 247 nm and two size groups of E153; 50 ± 10 nm and 950 ± 200 nm.

**Table 1:** Primary particle size, charge and specific surface area of E171 and E153

	DLS Mean (nm)	Particle size (TEM) Mean (nm)	Surface area (BET) m^2^/g	Zeta potential mV
E171	19 (1%)	135 ± 46 (27)	7.9	−37.2 ± 2.0
	880 ± 390 (94%)	305 ± 61 (12)		
	3200 (5%)	900 ± 247 (5)		
E153	283 ± 60	50 ± 10 (12)	1935	− 24.7 ± 1.6
		950 ± 200 (20)		

Dry particle size was measured by TEM analysis. Three size groups were observed from E171 and two size groups from E153. Surface area was determined by BET analysis. Zeta potential was determined by PALS. The data on TEM, surface area, and zeta potential are expressed as the mean ± SD (number of particles is indicated in the brackets). The hydrodynamic size distribution was also measured by DLS. Three size distributions were observed for E171. Numbers in brackets indicate the intensity distribution

The specific surface areas were 1935 and 7.9 m^2^/g for E153 and E171, respectively (Table 1). Aeroxide P25 (identical to NM105 in the European Commission’s Joint Research Centre) and Printex 90 have been thoroughly characterized in previous investigations [12, 13]. The specific surface areas are 295–338 and 46 m^2^/g for Printex 90 and Aeroxide P25, respectively.

The zeta potentials of the E171 and E153 particles in PBS were measured using PALS (phase analysis light scattering) and M3-PALS. E171 had a zeta potential of − 37.2 ± 2.0 mV and E153 of –24.7 ± 1.6 mV (Table 1). The results from single PALS measurements gave unusually high fluctuations; thus the hydrodynamic particle size was also measured with DLS on the same instrument. The DLS analysis showed a presence of microparticles (i.e. size of 6 μm and larger) and the suspension was not stable. The fluctuations in the zeta potential measurements might be caused by noise from aggregation of particles or sedimentation during the measurement.

**Fig. 2:**
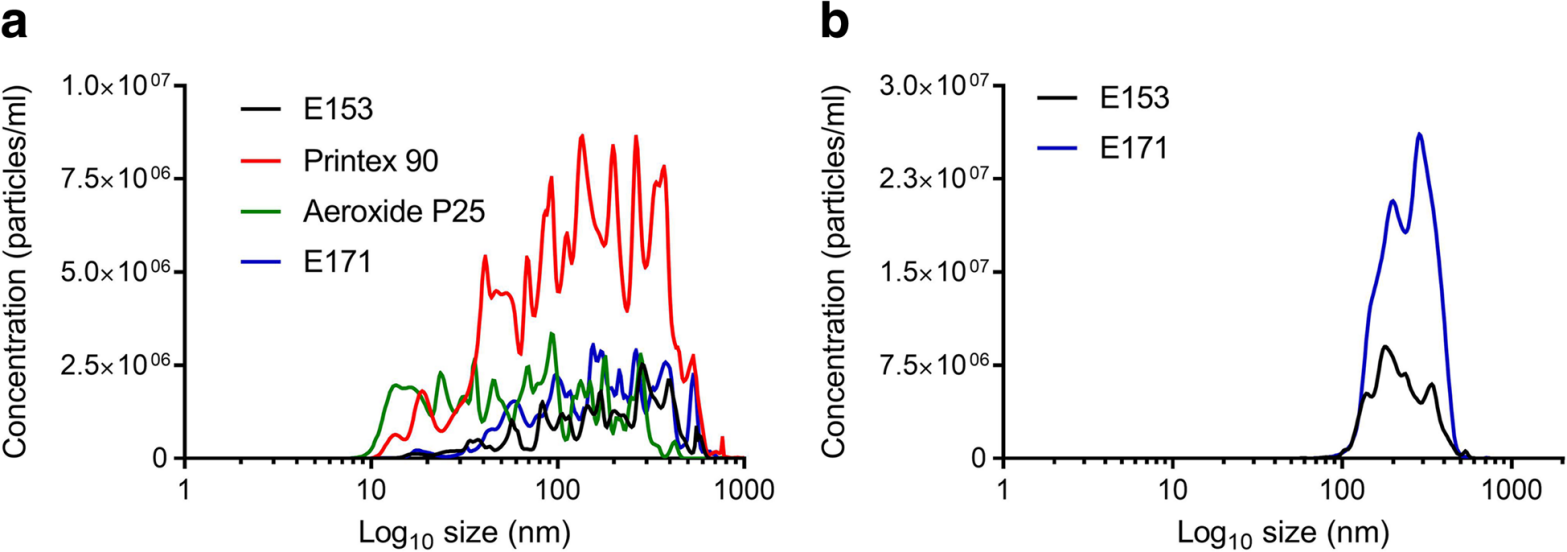
Hydrodynamic particle size distribution. The hydrodynamic size of the particles was determined with NanoSight LM20 and the Nanoparticle Tracking Analysis software 3.0. Particles used in the ex vivo study was dispersed in DMEM (**a**). Particles used in the in vivo study was dispersed in 0.45 μm filtered sterile water added 2% FBS (**b**). The data are presented as the mean particle size (nm) from three independent experiments. On each experimental day the mean of five following measurements were used

The hydrodynamic particle size distribution was measured by the Nanoparticle Tracking Analysis (NTA) (Table 2). Particles were suspended in DMEM in the ex vivo study and filtered water + 2% fetal bovine serum (FBS) in the in vivo study. Graphs of the hydrodynamic particle size distribution analysis are shown in Fig. 2. E171 and E153, dispersed in DMEM or filtered water, had a similar hydrodynamic particle size (E171: 203 ± 75 nm in DMEM and 270 ± 25 nm in filtered water; E153, 204 ± 58 nm in DMEM and 230 ± 24 nm in filtered water) (Table 2). Only 10% of the food-grade particles in DMEM suspensions were below 100 nm, whereas 50% of the benchmark particles (Aeroxide P25 and Printex 90) were below 100 nm.

**Table 2:** Hydrodynamic particle size of E171 and E153 in suspension vehicle for the ex vivo and in vivo studies

	NTA				
	Mean (nm)	Mode (nm)	D10 (nm)	D50 (nm)	D90 (nm)
Ex vivo Study					
E171	203 ± 75	264 ± 135	77 ± 69	179 ± 84	363 ± 126
Aeroxide P25	101 ± 58	128 ± 120	27 ± 17	79 ± 56	204 ± 121
E153	204 ± 58	291 ± 130	79 ± 45	192 ± 70	340 ± 112
Printex 90	166 ± 27	224 ± 119	35 ± 20	13 ± 20	339 ± 72
In vivo Study					
E171	270 ± 25	298 ± 58	163 ± 19	266 ± 28	374 ± 26
E153	230 ± 24	227 ± 61	137 ± 17	211 ± 24	347 ± 33

Hydrodynamic size distribution of particles used in the ex vivo and the in vivo study was measured by the Nanoparticle Tracking Analysis (NTA). The data are expressed as the mean, mode, distribution fractions D10, D50 and D90, ± SD. Each experiment was repeated three times on different days

### Ex vivo effect of food-grade and benchmark particles on the vasomotor function

Aorta rings from naïve rats were incubated for 30 min with E171 (14 μg/ml and 140 μg/ml), E153 (10 μg/ml and 140 μg/ml), Aeroxide P25 (14 μg/ml and 140 μg/ml) or Printex 90 (10 μg/ml and 100 μg/ml). We used a 30 min incubation period because it has been shown that ex vivo exposure to Printex 90 decreased ACh-induced vasorelaxation and increased the vasocontraction response to phenylephrine in aorta rings after exposure to 100 μg/ml for 30 min [11].

**Fig. 3:**
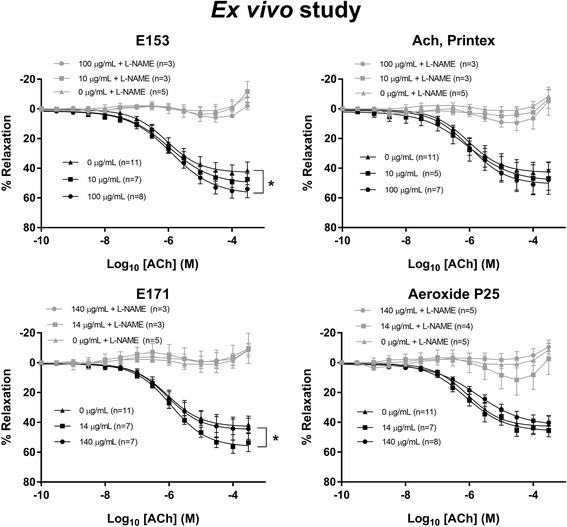
Acetylcholine (ACh)-induced endothelium-dependent vasorelaxation of rat aorta segments exposed ex vivo to particles. The measurements were performed with and without the addition of the NOS inhibitor, L-NAME. The acetylcholine response is expressed as the % relaxation of the pre-contraction tension produced by PGF_2a_. Each point on the curves represents the cumulative response at each concentration of acetylcholine. The data are presented as the mean ± SEM, *n* indicates the number of animals. An asterisk (*) denote a statistically significant effect on E_max_ compared to the control group (*P* < 0.05)

**Fig. 4:**
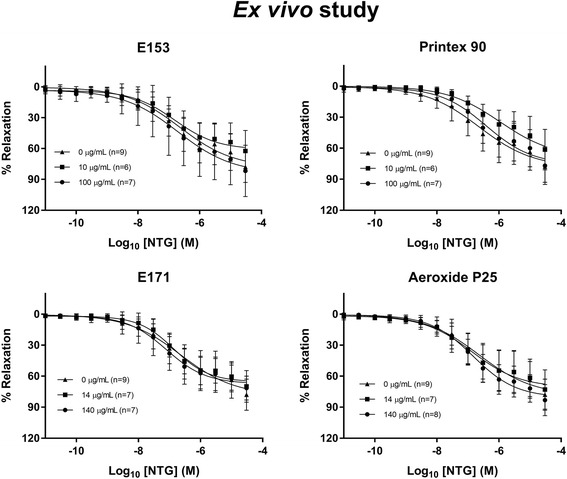
Nitroglycerine (NTG)-induced endothelium-independent vasorelaxation of rat aorta segments exposed ex vivo to particles. The nitroglycerine response is expressed as the % relaxation of the pre-contraction tension produced by PGF_2a_. Each point on the curves represents the cumulative response at each concentration of nitroglycerine. The data are presented as the mean ± SEM, *n* indicates the number of animals

**Fig. 5:**
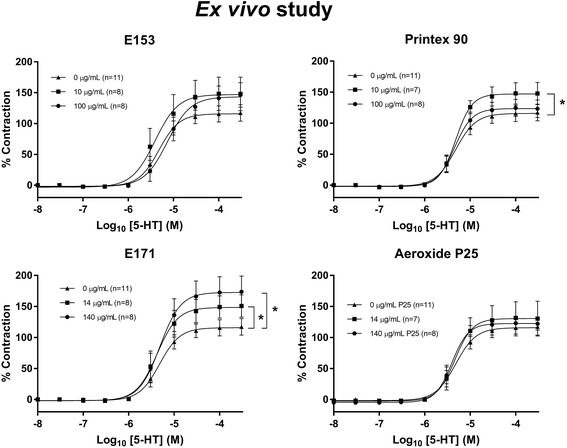
5-hydroxytryptamine(5-HT)-induced receptor-dependent vasocontraction of rat aorta segments exposed ex vivo to particles. The 5-HT response is expressed as the % maximal contraction induced by stimulation with K^+^ before the dose-response measurements. Each point on the curve represents the cumulative response at each concentration of 5-HT. The data are presented as the mean ± SEM, *n* indicates the number of animals. An asterisk (*) denote a statistically significant effect on E_max_ compared to the control group (*P* < 0.05)

The vessel rings were exposed to cumulative increasing concentrations of vasoactive compounds to assess ACh-induced endothelium-dependent vasorelaxation (Fig. 3), nitroglycerine (NTG)-induced endothelium-independent vasorelaxation (Fig. 4) or 5-hydroxytryptamine (5-HT)-induced vasocontraction (Fig. 5). Log EC_50_ and E_max_ values of the vasomotor responses are shown in Table 3. The analysis demonstrated a slightly increased maximal effect value (E_max_) of the ACh-dependent vasorelaxation in E171 and E153 exposed aorta rings (56.3%, 95% CI: 52.5–61.3%) and 57.5% (95% CI: 52.7–64.6%) respectively, compared to control group of 42.9% (95% CI: 38.7–48.6%) (Fig. 3). There were no differences in the NTG-mediated endothelium-independent vasorelaxation response (Fig. 4). As expected, co-incubation with *N*^G^-nitro-L-arginine methyl ester (L-NAME), an inhibitor of NOS, in the organ bath abolished the ACh-mediated vasorelaxation (Fig. 3). Thus, the results indicate an altered endothelium-dependent vasorelaxation response. The E171 and Printex 90 exposure also increased the E_max_ value of the 5-HT-mediated vasocontraction (Fig. 5). There was no effect on vasomotor when exposing rings with Aeroxide P25.

**Table 3:** Log EC_50_ and E_max_ values of concentration-response curves in näive aorta rings from wild-type rats

	Concentration (μg/ml)	Log EC_50_ (M)			E_max_ (%)		
		ACh	NTG	5-HT	ACh	NTG	5-HT
Control	0	−6.1 (− 6.3 to − 5.8)	− 6.6 (− 7.0 to − 5.7)	− 5.3 (− 5.5 to − 5.2)	42.9 (38.7–48.6)	78.3 (66.7–113.2)	115.8 (105.1–127.5)
E171	14	−5.9 (− 6.1 to − 5.7)	− 6.9 (− 7.1 to − 6.6)	− 5.4 (− 5.6 to − 5.2)	56.3* (52.5–61.3)	65.3 (59.9–73.5)	148.5* (131.3–168.2)
	140	−6.1 (− 6.4 to − 5.6)	− 7.1 (− 7.4 to − 6.7)	− 5.3 (− 5.5 to − 5.1)	44.9 (38.9–55.2)	67.2 (60.4–79.3)	172.7* (151.4–197.3)
Aeroxide P25	14	−6.1 (− 6.4 to − 5.8)	−6.9 (− 7.3 to − 6.1)	− 5.3 (− 5.6 to − 5.1)	45.9 (41.8–51.8)	70.8 (61.0–98.8)	130.6 (111.3–152.5)
	140	−5.7 (− 6.0 to − 5.3)	− 6.8 (− 7.0 to − 6.6)	−5.4 (− 5.5 to − 5.3)	42.4 (37.7–51.4)	80.5 (73.7–91.3)	122.5 (114.0–131.4)
E153	10	−6.0 (− 6.2 to − 5.7)	−6.8 (− 7.1 to − 6.2)	−5.4 (− 5.7 to − 5.1)	50.4 (45.9–57.5)	61.0 (53.3–79.6)	147.0 (122.7–179.5)
	100	−5.9 (− 6.1 to − 5.7)	− 6.7 (− 7.3 to 0.2)	−5.1 (− 5.3 to − 4.9)	57.5* (52.7–64.6)	84.1 (67.5–514.0)	143.4 (125.7–166.5)
Printex 90	10	−6.1 (− 6.7 to − 2.1)	−6.0 (− 6.5 to − 3.4)	− 5.3 (ND to − 5.2)	48.7 (39.3–161.2)	68.7 (53.0–188.8)	147.1* (134.8–160.0)
	100	−6.0 (− 6.3 to − 5.6)	−6.4 (− 6.8 to − 5.6)	−5.3 (− 5.5 to − 5.2)	50.8 (45.3–59.3)	75.6 (63.9–109.5)	123.6 (112.5–135.4)

The concentration-response curves were performed for acetylcholine (ACh), nitroglycerine (NTG) and 5-hydroxytryptamine (5-HT) on aorta from rats exposed ex vivo to E171, Aeroxide P25, E153 or Printex 90 for 30 min. The data are presented as the mean and 95% CI. The number of animals in each group varied; specified in Figs. 3, 4 and 5. Asterisk (*) denotes statistically significant effects on E_max_ when compared to the effect in the control group (*P* < 0.05), ND: the value could not be determined by non-linear regression analysis

### In vivo effect of food-grade particles on the vasomotor function in rats after intragastric administration of E171 and E153

**Fig. 6:**
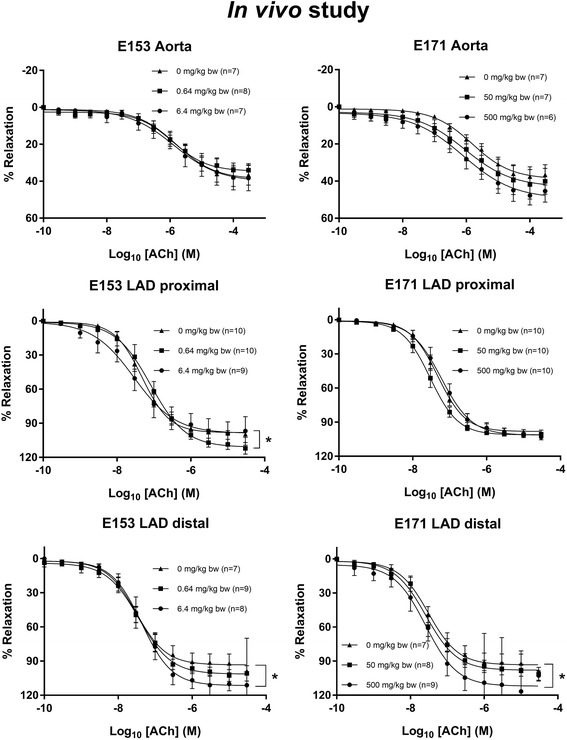
Acetylcholine (ACh)-induced endothelium-dependent vasorelaxation of artery segments from rats exposed intragastrically to particles. The acetylcholine response is expressed as the % relaxation of the pre-contraction tension produced by PGF_2a_. Each point on the curves represents the cumulative response at each concentration of acetylcholine. The data are presented as the mean ± SEM, *n* indicates the number of animals. An asterisk (*) denote a statistically significant effect on E_max_ compared to the control group (*P* < 0.05)

**Fig. 7:**
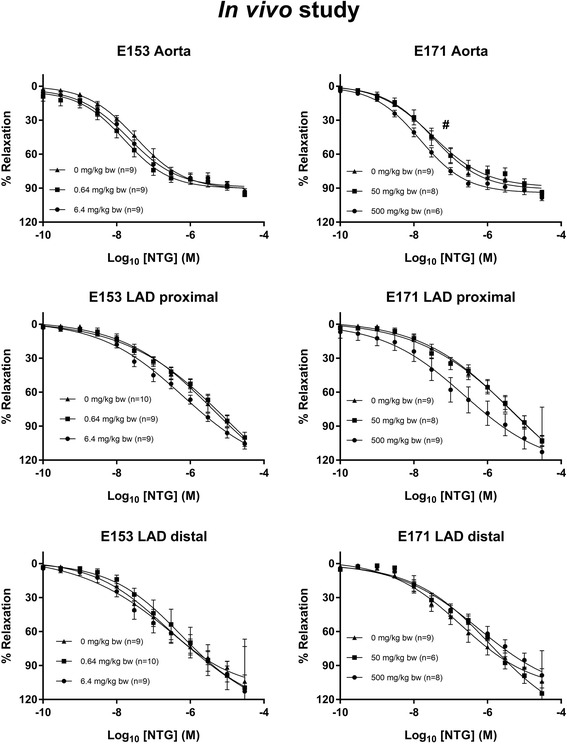
Nitroglycerine-induced endothelium-independent vasorelaxation of artery segments from rats exposed intragastrically to particles. The nitroglycerine response is expressed as the % relaxation of the pre-contraction tension produced by PGF_2a_. Each point on the curves represents the cumulative response at each concentration of nitroglycerine. The data are presented as the mean ± SEM, *n* indicates the number of animals. # denotes a statistically significant effect on log EC_50_ compared to the control group (*P* < 0.05)

**Fig. 8:**
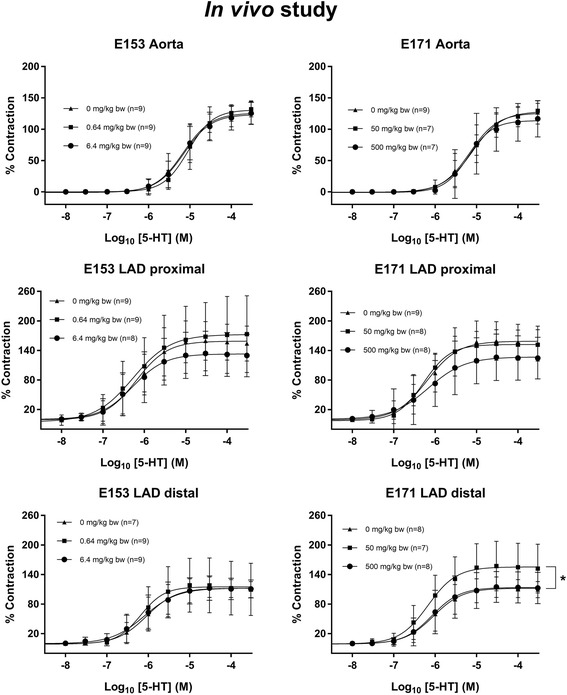
5-hydroxytryptamine (5-HT)-induced receptor-dependent vasocontraction of artery segments from rats exposed intragastrically to particles. The 5-HT response is expressed as the % maximal contraction induced by stimulation with K^+^ before the dose-response measurements. Each point on the curve represents the cumulative response at each concentration of 5-HT. The data are presented as the mean ± SEM, *n* indicates the number of animals. An asterisk (*) denote a statistically significant effect on E_max_ compared to the control group (*P* < 0.05)

The vasomotor function in coronary arteries and aorta were assessed in rats after intragastric administration of E171, E153 or vehicle, once a week for 10 weeks. The Log EC_50_ and E_max_ values are shown in Table 4. The maximal ACh-mediated endothelium-dependent vasorelaxation response was slightly increased in the proximal left anterior descending (LAD) artery of the low-dose E153 exposed rats (111.1%, 95% CI: 104.3–119.1%) compared to the control group of 98.2% (95% CI: 94.5–102.0%) (Fig. 6). There was also increased maximal ACh-mediated endothelium-dependent vasorelaxation response in the distal LAD in the high-dose E153 exposed rats (111.5%, 95% CI: 103.5–120.6%) compared to the control group of 93.3% (95% CI: 87.1–100.4%), and in the high-dose E171 exposed rats (112.0%, 95% CI: 101.9–124.7%), whereas there was no difference in the aorta (Fig. 6). The E_max_ value of the NTG-mediated endothelium-independent vasorelaxation response was unaffected (Fig. 7). The LogEC_50_ of the E171 high-dose exposed rats was shifted to the left (− 7.8, 95% CI: -8.0 to − 7.7) compared to the control (− 7.5, 95% CI: -7.6 to − 7.4). This effect was not observed in any of the other exposures and segments. Overall, the findings on the NTG- and ACh- mediated responses suggest that the altered ACh-mediated vasorelaxation response is indeed an endothelium-dependent effect on the vasomotor function. The 5-HT-mediated vasocontraction response was increased in the distal (50 mg/kg) LAD (Fig. 8).

**Table 4:** Log EC_50_ and E_max_ values of concentration-response curves in aorta and left anterior descending coronary artery (LAD)

	Dose (mg/kg bw/week)	Log EC_50_ (M)			E_max_ (%)		
		Aorta	LAD proximal	LAD distal	Aorta	LAD proximal	LAD distal
ACh							
Control	0	−5.8 (−6.1 to −5.4)	−7.3 (−7.4 to − 7.2)	−7.5 (− 7.7 to − 7.3)	38.9 (34.6–46.0)	98.2 (94.5–102.0)	93.3 (87.1–100.4)
E171	50	− 6.0 (− 6.7 to − 2.3)	− 7.5 (− 7.6 to − 7.4)	−7.6 (− 7.7 to − 7.4)	43.1 (35.2–131.6)	101.1 (97.6–104.7)	98.0 (92.3–104.5)
	500	−6.1 (− 6.6 to − 5.1)	−7.2 (− 7.4 to − 7.1)	−7.6 (− 7.9 to − 7.3)	49.5 (42.4–75.0)	101.4 (96.1–107.4)	112.0* (101.9–124.7)
E153	0.64	−5.9 (− 6.2 to − 5.5)	−7.1 (− 7.2 to − 6.9)	−7.4 (− 7.6 to − 7.3)	34.6 (30.7–41.4)	111.1* (104.3–119.1)	101.4 (95.9–107.6)
	6.4	−5.9 (− 6.4 to − 4.3)	−7.5 (− 8.0 to − 7.2)	−7.4 (− 7.6 to − 7.2)	40.2 (33.2–74.1)	98.9 (88.5–116.1)	111.5* (103.5–120.6)
NTG							
Control	0	−7.5 (− 7.6 to − 7.4)	− 5.4 (− 6.1 to − 3.0)	−6.7 (− 7.1 to − 6.1)	91.0 (87.2–95.6)	153.1 (115.7–365.2)	111.1 (96.8–144.3)
E171	50	−7.5 (− 7.7 to − 7.2)	− 5.2 (− 6.0 to − 0.1)	−5.9 (− 6.4 to − 4.7)	89.0 (82.2–99.3)	166.2 (118.7–902.1)	143.0 (117.2–226.7)
	500	−7.8* (− 8.0 to − 7.7)	− 6.6 (− 7.3 to 2.6)	− 6.2 (− 6.7 to − 4.9)	94.1 (90.5–98.2)	125.8 (98.9–1086.0)	115.3 (94.8–188.8)
E153	0.64	−7.8 (− 8.0 to − 7.6)	− 5.0 (− 5.9 to 0.8)	−6.3 (− 6.9 to − 6.1)	90.2 (85.1–96.8)	176.0 (122.9–1208.0)	126.4 (98.3–480.6)
	6.4	−7.7 (− 7.9 to − 7.5)	−6.2 (− 6.6 to − 5.5)	−6.2 (− 7.0 to 17.1)	89.2 (83.8–96.3)	129.5 (111.4–171.2)	142.5 (107.8–26,498.0)
5-HT							
Control	0	−5.2 (− 5.3 to − 5.0)	− 6.2 (− 6.4 to − 6.0)	− 6.0 (− 6.2 to − 5.9)	125.8 (117.7–135.4)	158.9 (147.5–171.5)	112.2 (104.4–120.9)
E171	50	−5.1 (− 5.2 to − 5.0)	−6.3 (− 6.4 to − 6.1)	−6.2 (− 6.4 to − 6.0)	128.4 (120.7–137.8)	152.2 (144.1–160.7)	155.4* (141.5–171.3)
	500	−5.2 (− 5.4 to − 5.0)	−6.2 (− 6.6 to − 5.8)	−6.1 (− 6.3 to − 5.8)	113.9 (100.4–132.5)	126.9 (111.5–149.5)	113.7 (102.6–126.7)
E153	0.64	−5.0 (− 5.1 to − 4.9)	− 6.3 (− 6.6 to − 5.9)	−6.2 (− 6.4 to − 5.9)	130.8 (122.2–141.1)	172.4 (152.6–201.3)	115.1 (102.6–128.7)
	6.4	−5.1 (− 5.3 to − 5.0)	− 6.3 (− 6.6 to − 6.0)	6.1 (− 6.3 to − 5.9)	123.5 (115.2–133.6)	132.8 (119.4–148.9)	112.2 (103.9–121.9)

The concentration-response curves were performed for acetylcholine (ACh), nitroglycerine (NTG) and 5-hydroxytryptamine (5-HT) on the aorta and coronary arteries from rats exposed intragastrically to E171 or E153 once a week for 10 weeks. The data are presented as mean and 95% CI. The number of animals in each group varied; specified in the concentration-response curves (Figs. 6, 7 and 8). An asterisk (*) denotes statistically significant effects when compared to the effect in the control group (*P* < 0.05)

### Biochemical parameters in plasma of rats after intragastric administration of E171 and E153

Plasma levels of vitamin C (calculated as ascorbate + dehydroascorbic acid (DHA; an oxidized form of ascorbate)), BH_4_ and the oxidized form dihydrobiopterin (BH_2_), asymmetric dimethylarginine (ADMA) and L-arginine (L-Arg) were measured to assess systemic oxidative stress and imbalances in the NO bioavailability. ADMA competes with arginine as a substrate for eNOS and functions as an endogenous eNOS inhibitor. Increased plasma levels of ADMA have been observed in rats after exposure to concentrated fine ambient air particles [14]. Malondialdehyde (MDA) is a lipid peroxidation product and was measured as a marker of oxidative stress.

In general, the plasma biomarkers were unaltered in particle-exposed rats as compared to controls (Table 5). Collectively, the results on plasma antioxidants (ascorbate and BH_4_) and oxidants (i.e., MDA, and BH_2_) demonstrated no change in systemic oxidative stress in response to the particle exposure in rats.

**Table 5:** Biochemical parameters in lean Zucker rats after repeated exposure to E171 and E153 by oral gavage

In vivo Study	Control (*n* = 9)	E171 (n = 9)	E171 (*n* = 10)	E153 (n = 10)	E153 (*n* = 8)
Dose (mg/kg bw/week)	0	50	500	0.64	6.4
Vitamin C (μM) (ascorbate + DHA)	19.6 ± 2.9	20.3 ± 2.6	21.0 ± 4.6	20.5 ± 3.9	18.9 ± 2.9
MDA (μM)	0.20 ± 0.05	0.19 ± 0.08	0.22 ± 0.07	0.20 ± 0.07	0.19 ± 0.05
BH_4_ (nM)	150.1 ± 20.4	149.2 ± 18.9	154.8 ± 43.7	151.5 ± 20.5	134.5 ± 27.4
BH_4_/BH_2_ ratio (nM)	0.20 ± 0.04	0.18 ± 0.04	0.19 ± 0.04	0.17 ± 0.05	0.21 ± 0.05
ADMA (μM)	178.1 ± 31.8	192.3 ± 41.0	179.1 ± 47.0	219.8 ± 118.1	215.1 ± 89.4
L-arginine (μM)	51.0 ± 8.8	61.8 ± 15.8	58.3 ± 16.9	75.5 ± 54.1	75.1 ± 35.5

All biochemical parameters were measured in plasma samples taken at the time of sacrifice. bw: body weight, *DHA* dehydroascorbic acid, *MDA* malondialdehyde, *BH4* tetrahydrobiopterin, *BH2* dihydrobiopterin, *ADMA* asymmetric dimethylarginine. Data are represented as mean ± SD

## Discussion

The results of this study show that in vivo exposure to food-grade TiO_2_ (E171) and vegetable carbon (E153) was associated with a slightly altered endothelium-dependent vasorelaxation and vasocontraction response in coronary arteries. The same effects were observed in aorta rings from naïve rats after direct exposure to E171 and E153. This consistency in ex vivo and in vivo responses should be noted, although it is equally important to note that the effects on vasomotor function did not occur in a dose-dependent manner. The study represents an exposure to “real-life” particles in the relevant route of exposure in humans after ingestion of either food or pharmaceuticals. In general, the exposure to E153 and E171 is higher in children than adults and elderly [9, 10]. Nevertheless, the elderly, patients with pre-existing diseases and adults with cardiometabolic risk factors may be more suspectible to particle-induced cardiovascular disease than children and adolescents.

A systematic review has demonstrated that the majority of studies on particle exposure in animals and humans show reduced endothelium-dependent vasorelaxation and increased vasocontraction response in arteries [15]. The immediate consequence of this effect is an increased tone of the artery, which might be associated with elevated blood pressure. Thus, the increase in both the vasorelaxation and vasocontraction response, as found in the present study, is somewhat surprising, although other studies have demonstrated similar responses. It has been observed that amorphous nano silica particles induced vasorelaxation in thoracic aorta rings of rats ex vivo, which was mediated through NO by activation of PI3K/Akt/eNOS signaling [16]. Earlier studies on high-dose pulmonary exposure to ambient particulate matter also showed increased ACh-dependent vasorelaxation in aorta rings from spontaneously hypertensive rats [17]. The same type of exposure to ambient particulate matter provoked vasorelaxation in aorta rings after ex vivo exposure [18, 19]. In our study, the increased vasorelaxation after ACh exposure to vessels may originate from an eNOS-dependent pathway since L-NAME abolished the effect in the ex vivo study and the NTG-mediated vasorelaxation was unaltered. It is possible that the concurrent increase in both vasorelaxation and vasocontraction occur by different mechanisms, although they may also be the product of compensation in the vessel. The effect on vasomotor function was seen in resistance arteries, whereas the aorta (i.e., a conductance artery) was not affected. The endothelium-dependent vasorelaxation in conductance arteries is mainly driven by NO, whereas resistance arteries also use endothelium-derived hyperpolarization factor [20].

The effects on vasomotor function responses in the in vivo study were in the range of 20–40% difference in the distal LAD. To the best of our knowledge, there are no observations on vasomotor function in humans after oral exposure to particles. It has been shown that gastric exposure to nanosized CeO_2_ produced a smaller impairment of microvascular function in Sprague-Dawley rats compared to the same dose administered by intratracheal instillation [21]. However, a larger induction of oxidatively damaged DNA has been observed in the liver after oral exposure to Printex 90 compared to the same dose administered by intratracheal instillation to Fisher F344 rats [22]. Previously, we have obtained less than 10% difference in microvascular function in humans after controlled inhalation exposure to particulate matter and the effect has been strongest in risk groups of cardiovascular disease and elderly [23–29]. The magnitude of the effects on vasomotor function in the present study is modest, but they are also realistic, considering the magnitude of exposure.

The toxicity of nanosized particles is typically considered to be related to inflammation and oxidative stress. We chased this mechanism by analysis of plasma levels of the lipid peroxidation products (MDA) and antioxidants (ascorbate and BH_4_). However, these results did not indicate a systemic oxidative stress reaction. It is important to emphasize that MDA was measured with a validated HPLC assay. Likewise, care was taken to secure plasma from spurious oxidation during sampling and processing for the ascorbate and BH_4_ assays. It should be noted that some studies have shown associations between exposure to particles and altered vasomotor function without systemic inflammation and oxidative stress [3]. Still, oxidative stress and inflammation may occur in the vascular wall as shown in a study where inhalation of TiO_2_ generated microvascular dysfunction, concurrently with increased production of reactive oxygen species and nitrosative stress [30]. Unfortunately, the lack of oxidative stress indicators in plasma leaves the study without a link in the causal pathway from external exposure to the observed vasomotor function response in the in vivo study. Nevertheless, we speculate that the altered vasomotor response may be due to bioactive components in plasma that interact with the cellular signaling (e.g., NO production or delivery from endothelial cells to smooth muscle cells) or increases the response to vasoactive drugs. Previously, we have shown that plasma from Printex 90 exposed mice caused vasocontraction in aorta rings from naïve mice [31]. This type of bioactivity has been described in various studies on pulmonary exposure to particulate matter [32]. Unfortunately, we did not have sufficient plasma to measure plasma bioactivity.

The ex vivo experiments indicated that E171 and E153 could generate the same effects as observed in vivo after oral exposure. In a previous study, we observed that fine, nanosized and photocatalytic TiO_2_ increased the surface expression of cell adhesion molecules in human umbilical vein endothelial cells without concurrent generation of reactive oxygen species [33]. We used Printex 90 as a benchmark particle, which previously demonstrated increased phenylephrine-mediated vasocontraction in mice aorta rings following exposure to 100 μg/ml [11]. The ACh-vasorelaxation response was mixed in the sense that it was associated with a decreased response at 10 μg/ml and increased response at 100 μg/ml [11]. However, it should be noted that the exposure conditions were different as the aorta rings were exposed directly in the organ bath in the mouse study while in the present study, rat aorta rings were exposed to particles before they were mounted in the organ bath. Also, there is a clear difference in species. In our previous study, we also exposed rat mesenteric arteries to Printex 90 in a pressure myograph, which demonstrated a decrease in the vessel diameter, indicating an overall vasocontraction response to Printex 90.

We did not measure cytotoxicity in the ex vivo study because the exposure only lasted 30 min, which is too short to detect a reliable response by conventional techniques such as the lactate dehydrogenase activity and WST-1 assays. In a previous study we did not observe statistically significant increases in cytotoxicity in human umbilical vein endothelial cells (HUVECs) after 3 or 24 h exposure to Printex 90 [34]. Equivocal results on Printex 90 in HUVECs were observed after 24-h exposure periods as there was 20% increase in lactate dehydrogenase (LDH) activity assay, whereas the WST-1 assay was unaltered after exposure to 100 μg/ml) [11]. On the other hand, a fine size carbon black (Flammrus 101) did not generate cytotoxicity in the LDH assay [33]. TiO_2_ has low toxicity as compared to other nanomaterials [35]. We have obtained mixed results with TiO_2_ in HUVECs; one study showed no effect of fine and photocatalytic TiO_2_ particles, whereas a nanosized sample generated approximately 20% cytotoxicity (LDH assay) [33]. Another study actually showed a rather large effect in the WST-1 assay after exposure to 7 nm anatase TiO_2_ and some rutile forms [36]. It should be noted that HUVECs are adherent cells and the exposure may increase during the incubation period because of sedimentation of particles, whereas the aorta rings were exposed in suspension in the present study. In addition, there is no consensus about the role of cytotoxicity in ex vivo studies on vasomotor function. In genotoxicology, it is recommended that the level of cytotoxicity should not increase more than 30% in assays for DNA damage such as the comet assay [37].

There is no consistency in the available literature concerning the uptake of TiO_2_ to the circulation [38]. Earlier studies on micro size (500 nm) TiO_2_ in rats demonstrated a relatively high fraction of absorption (approximately 6.5% of the administered dose) after exposure by oral gavage to 12.5 mg/kg per day for 10 days [39]. However, a recent study demonstrated only 0.6% absorption after a single small dose of radioactively labeled 70 nm TiO_2_ nanoparticles in rats [40]. E171 mainly consists of micro-sized TiO_2_ particles, with less than 3.2% of the mass in the nanoscale range [10]. Taking the dose level and particle size into consideration, the exposure in our study may be more similar to the observations of translocation of micro-sized particles in rats.

Nevertheless, it appears safe to assume that the translocation is low. Uptake of TiO_2_ to the systemic circulation has been described in humans [41]. It is even more unclear whether carbon-based particles translocate to the systemic circulation because it is more difficult to measure carbon material than metals in tissues.

## Conclusion

This study shows that 10 weeks of intragastric exposure to vegetable carbon (E151) or food-grade TiO_2_ (E171) was associated with modest albeit statistically significant alterations in the vasomotor response in coronary arteries, without evidence of systemic oxidative stress.

## Methods

### Particles

E153 (Norit N.V., Amersfoort, The Netherlands, distributed by Medic Team A/S, Allerød, Denmark) was purchased as an over-the-counter drug in a local pharmacy. Each package contained 30 gelatine capsules with 200 mg vegetable carbon powder. Norit is marketed as a drug for treatment of acute poisoning and diarrhea; it is commonly described as “activated charcoal” or “activated carbon”. The gelatine capsule was discharged, and the internal powder content was stored in a sterile container. Food-grade TiO_2_ was bought as a white coloring agent (E171) at a Danish webshop (www.bolsjehuset.dk). Aeroxide P25 was obtained from Sigma-Aldrich (product number 718467, declared primary particle size of 21 nm). Printex 90 was kindly supplied from Degussa-Hüls, Frankfurt, Germany (declared primary particle size of 14 nm).

We characterized E171 and E153 with both transmission electron microscopy and specific surface areas. The latter used nitrogen adsorption according to the Brunauer, Emmet and Teller method [42]. The analysis was performed at Quantachrome Instruments (LabQMC, Boynton Beach, FL, USA). Particles were suspended in water with 2% FBS in a concentration of 100 μg/ml for the visualization in TEM. A few drops of the suspension were applied on TEM grids and dried [43]. Afterwards the TEM grids were inspected in a Phillips CM20 transmission microscope at 5800 and 44,000 magnification. We measured the size of individual particles using ImageJ software (https://imagej.nih.gov) on digital images from two different grids, which were prepared on the same day of analysis.

Zeta potential was measured on a Brookhaven ZetaPALS (Brookhaven Instruments, Holtsville NY, USA) and a Zetasizer Nano ZS (Malvern Instruments, Malvern, UK). E171 and E153 were sonicated for 30 min at 60 Hz using a VWR UltraSonic Cleaner (VWR, Radnor PA, USA) and diluted to 0.2 mg/mL in PBS (P4417, Sigma). Measurements were on both instruments performed using a dip cell. On the ZetaPALS instrument, ten runs on three different particle suspensions were done, on the Zetasizer, three runs were done on the same samples. Reported values in Table 1 are from the Zetasizer, the encountered fluctuations in signal were observed on the ZetaPALS.

The hydrodynamic particle size in suspension was determined by the Nanoparticle Tracking Analysis (NTA). The particles were sonicated as described below and diluted to a final concentration of approximately 10 μg/ml. The samples were analyzed on a NanoSight LM20 (Nanosight Ltd., Malvern Instruments, Malvern, UK) with a blue (450 nm) laser. All measurements were repeated on three different days. On each experimental day, five consecutive measurements on the same particle suspension were assessed, and the mean was used for data analysis.

For the ex vivo and in vivo study particle stock suspensions were prepared by dispersing particles in either 0.45 μm filtered sterile water added 2% FBS (ref. 10,500–064, Gibco®) (in vivo study) or DMEM cell culture medium (Dulbecco’s modified eagle medium, D5796, Sigma) (ex vivo study), using a Branson Sonifier mounted with a disruptor horn (Model S-450D, Branson Ultrasonics Corp., Danbury, CT, USA) in the in vivo study or using a Vibra Cell Vc50t, Ultrasonic Processor 20 kHz (R125136, Sonics & Materials, Newtown, Connecticut, USA) in the ex vivo study. Stock suspensions were sonicated for 16 min without pause and continuously cooled on ice, according to the recommendations in the protocol that was developed for the EU Framework 7 project “Risk assessment of engineered nanoparticles” (ENPRA; http://www.enpra.eu). Stock suspension concentrations were in the ex vivo study 1.4 mg/ml for E171 and Aeroxide P25 and 1.0 mg/ml for E153 and Printex 90. Stock suspensions for the in vivo study were 120 mg/ml for E171 and 1.6 mg/ml for E153. The stock solutions were prepared the same day of use and used immediately after sonication (vortexed before use) after dilution with dispersion media to final concentrations.

### Animal model and study design

#### Ex vivo study

Eleven female Sprague-Dawley rats were purchased from Taconic (Ejby, Denmark). The rats were aged between 14 and 17 weeks when sacrificed and weighed 297 ± 21 g. The rats were acclimatized for minimum 1 week before entering the study. The rats were housed in pairs, in an enriched environment, with free access to tap water and standard chow. The cages were housed in an animal facility with a 12 h day-night cycle in a temperature (20–24 °C) and moisture (55%) controlled room. At the day of the experiment, one rat was sacrificed. The rat was anesthetized with a subcutaneous injection (in the lower back) of a combination of hypnorm (fentanyl 0.315 mg/ml and fluanisone 10 mg/ml) and dormicum (midazolam, 5 mg/ml) (0.3 ml/100 g bw), and decapitated following deep anesthesia and concomitant disappearance of voluntary reflexes. The heart with the aorta was isolated and put in a glass vial containing ice-cold PSS buffer, until dissection in the laboratory facilities immediately thereafter. PSS had the following composition: 119.0 mM NaCl, 4.7 mM KCl, 1.5 mM CaCl_2_, 1.2 mM MgSO_4_, 1.2 KH_2_PO_4_ mM, 0.027 EDTA mM, 6.05 mM glucose, 25.0 mM NaHCO_3_. pH was 7.4 when the solution was gassed with 95% O_2_ and 5% CO_2_ mixture. The aorta was dissected free of fat- and connective tissue in a petri dish containing ice cold PSS and cut into segments of approximately 2 mm in length and incubated for 30 min in particle suspension (DMEM) (in a 12-well plate/2 ml per well) at 37 °C with 5% CO_2_. Particles and concentrations were either E171 (14 or 140 μg/ml), Aeroxide P25 (14 or 140 μg/ml), E153 (10 or 100 μg/ml) or Printex 90 (10 or 100 μg/ml). After the exposure to particles, the aorta rings were carefully washed in a physiological saline solution (PSS) and mounted in a wire myograph. Each segment was normalized to a standard pressure of 13.3 kPa, and an internal circumference (IC) set to 90% (IC_90_) of the IC_100_ (pressure at 100 mmHg). After a 30 min equilibration period, the rings were stimulated with a high potassium concentration to test their viability and then preconstricted with prostaglandin F_2α_ (PGF_2α_) before vasorelaxation studies. There was no difference between the precontraction ability of aorta rings that had been exposed to particles or vehicle (results not shown). The rings were challenged with vasoactive compounds as described below.

#### In vivo study

Lean Zucker rats were used because of previous findings of endothelial dysfunction in aorta rings in this strain after exposure to Printex 90 [6]. The study showed similar vasomotor dysfunction response in both lean and obese Zucker rats, and therefore the less expensive lean Zucker rat was chosen as a model animal in this study. The obese Zucker rat is an animal model for metabolic syndrome, and it develops vasomotor dysfunction [6, 44].

Fifty, female lean Zucker rats (Crl:ZUC-Lepr^fa^, strain code 138), were purchased from Charles River (distributed in Denmark by Scanbur, Karlslunde, Denmark). The rats were between 8 and 13 weeks of age and acclimatized for at least 1 week before entering the study. All rats were randomly assigned to one of five exposure groups; vehicle, low-dose E153 (0.64 mg/kg bw/week), high-dose E153 (6.4 mg/kg bw/week), low-dose E171 (50 mg/kg bw/week) or high-dose E171 (500 mg/kg bw/week). Each exposure group included ten rats. The group size was based on a power calculation of previous results on vasomotor function in the aorta of Printex 90 exposed lean Zucker rats [6] (calculations not shown). The rats were housed in pairs in cages with standard bedding and nesting materials. Rats, living in the same cage, were assigned to the same exposure, to avoid the possibility of cross-contamination of particles via, e.g., ingestion of stool pellets. The rats had unlimited access to standard rodent chow (Altromin no. 1319) and tap water. None of the rats were euthanized before the end of the exposure period due to discomfort, assessed as excessive loss of body weight (exceeding 20% over a week). The bodyweights before (188 ± 20 g) and after (239 g ± 15 g) the exposure period were not different between groups (mean and standard deviation). Particles were administered to the rat by oral gavage of 1 ml particle suspension or vehicle, with the use of a plastic feeding tube (15ga × 100 mm, Instech Laboratories, Winsum, the Netherlands). The rats were weighed the day before exposure, and the particle stock suspension was adjusted to individual weight. Control rats received a bolus of 1 ml dispersion media, sonicated as described above, but without particles. Animals were exposed once a week, for 10 weeks. Oral gavage was executed on the same weekday and time a day for each animal. The procedure was performed by an experienced animal technician to minimize stress and discomfort in the rats. At the day of the experiment, two rats, from different exposure groups, was sacrificed. The rat was anesthetized 24 h after the last exposure, with a subcutaneous injection (in the lower back) of a combination of hypnorm (fentanyl 0.315 mg/ml and fluanisone 10 mg/ml) and dormicum (midazolam, 5 mg/ml) (0.3 ml/100 g bw), following death by exsanguination due to blood collection.

### Biochemical parameters in plasma

Blood was collected by abdominal aortic puncture using a 21 gauge needle equipped with a syringe (without anticoagulant). Approximately 4 ml blood was immediately transferred to an ethylenediamine-tetraacetic acid (EDTA) K3 tube (2.1 mg/ml EDTA K3, Venosafe®) and plasma was collected after centrifugation (2000 g, 4 °C for 5 min). The remaining blood was set to coagulate in an Eppendorf tube, and serum was collected by centrifugation (2000 g, 4° for 5 min). Plasma from EDTA treated blood was used to measure concentrations of MDA and ADMA. For measurement of BH_4_, EDTA treated blood was added 4% dithioerythritol and plasma were collected by centrifugation (1500 g, 4 °C for 1 min). For measurement of vitamin C, 10% metaphosphoric acid was added to plasma from EDTA tubes and stored at − 80 °C until analyzed. Plasma concentrations of BH_4_, vitamin C, MDA, and AMDA were measured by HPLC with fluorescent detection [45–48].

### Vasomotor function assessed by the wire myograph method

The procedure for assessing vasomotor function was similar in the ex vivo- and the in vivo study. In the ex vivo study, segments of the aorta was incubated with particles for 30 min and then assessed for vasomotor responses. In both the ex vivo- and in vivo study segments of the aorta was used. In the in vivo study, we also chose to include segments of both the proximal and distal segments of the LAD. This was due to possible differences in vasomotor responses in LAD segments compared to the aorta. LAD mainly resembles resistance arteries whereas the aorta is a conductance artery, in addition, coronary arteries are a common site for atheroma formation. The vasomotor function was analyzed immediately after the rats were sacrificed and blood was collected. The heart with aorta was excised from the rats and kept in ice-cold PSS before dissection. Before dissection, the heart and aorta were secured gently with entomology pins in a petri dish with a thick layer of clear silicone gel in the base. The tissue was covered with Ca^2+^free PSS buffer. This buffer had the same composition as PSS, only CaCl_2_ was omitted, and 1 μM ethylene glycol tetraacetic acid was added. The dissection was done in Ca^2+^free PSS buffer to avoid unnecessary vessel contraction. The aortic root and arch were gently trimmed free of connective- and fat tissue under a dissecting microscope. One segment of the ascending aorta cut immediately before the three large side branches of the aortic arch, was cut into a ring-shaped segment of approximately 2 mm in length. The vessel was carefully cannulated with two stainless steel wires (40 μm in diameter) and mounted in the organ bath of a Multi-Wire Myograph 620 M (Danish Myo Technology, Aarhus, Denmark) interfaced to a PowerLab4/35 recorder (ADInstruments). The organ bath was filled with cold, oxygenated Ca^2+^free PSS buffer (5 ml) and continuously perfused with a 95% O_2_ and 5% CO_2_ gas mixture. Perfusion with the gas mixture was continuously done throughout the entire experimental procedure. In the in vivo study, we also included two segments of the LAD artery. The LAD artery was dissected free from the heart by carefully removing a part of the myocardial tissue. One segment of the LAD proximal to the aortic root, and one segment distal to the aortic root (nearer the apex) were cut into ring-shaped segments of approximately 2 mm in length. The LAD segments were mounted in organ baths as described for the aorta but cannulated with a stainless steel wire of 25 μm in diameter. When finished mounting, Ca^2+^free PSS buffer was removed from the organ bath, and replaced by cold PSS. The heat was turned on to 37 °C, and the segments were allowed to equilibrate for 30 min. All baths were maintained at 37 °C throughout the experiment. The DMT LabChart normalization procedure was used, to set vessels to standard initial conditions. Normalization was done to ensure that the physiological responses of the vessels were assessed reliably. With normalization, the internal circumference a vessel would have if relaxed and under a transmural pressure of 100 mmHg (IC_100_), is determined. The maximal active tension was set to 90% (IC1) of IC_100_, which is optimal at this point. After normalization, the vessel viability was confirmed by the use of a “wake-up” protocol. The protocol consisted of stimulation with 37 °C potassium physiological salt solution (KPSS). KPSS had the same constituents as PSS but without NaCl and the concentration of KCl was 125 mM. KPSS induces non-receptor mediated vasocontraction, due to depolarization of the cell by enhancing extracellular influx of calcium. The contraction was allowed to stabilize and reach a plateau before adding PSS. The stimulation was repeated three times, and only responsive vessels were used in the further experiments. ACh (acetylcholine chloride, Sigma Aldrich) was used to assess the endothelium-dependent vasorelaxation in PGF_2α_ (3–10 μM) (dinoprost trometamol 5 mg/mL, Glostrup apotek, Denmark) precontracted vessels. The particle exposure did not alter the precontraction ability with PGF_2α_ (results not shown). ACh was cumulatively added to the organ bath (0.1 nM - 0.3 mM in aorta, 0.1 nm - 0.03 mM in LAD), each concentration was allowed to reach a plateau before adding the next concentration. To assess if the ACh-induced relaxation were dependent on eNOS, some of the arteries in the ex vivo study (*n* = 3–5) were incubated with the NOS inhibitor L-NAME (N5751, Sigma-Aldrich) for 15 min (10^− 5^ M), before an additional concentration-response curve with acetylcholine were assessed.

To assess the endothelium-independent vasorelaxation, the NO donor nitroglycerin (NTG) (glyceryl nitrate 5 mg/mL, Region H’s Apotek, Herlev, Denmark) was added to the organ bath in a cumulative manner (ex vivo: 0.01 nm - 0.03 mM; in vivo: 0.1 nm 0.03 mM) after inducing a stable contraction in the vessels with PGF_2α_. The arteries responses to receptor-dependent vasocontraction were assessed with 5-hydroxytryptamine (5-HT, serotonin hydrochloride, H9523, Sigma Aldrich) (ex vivo: 10 nm - 0.3 mM; in vivo: 3.0 nm 0.3 mM).

Between the cumulative concentration-response curves with ACh, NTG, and 5-HT, the vessels were washed with PSS and contracted once with KPSS. The precontraction response with PGF_2α_ was expressed as the percentage of the maximal contraction when stimulating the vessels with K^+^ (125 mM). Vessels precontracted to 10–150% of the maximal K^+^ induced contraction was included in the study. There was no difference between groups in the precontraction response. The vasorelaxation caused by ACh and NTG were expressed as the percentage relaxation of the precontraction tension produced by PGF_2α_. The contraction caused by 5-HT were expressed as the percentage of the maximal contraction obtained when stimulating the vessel segments with K^+^ (125 mM) before the 5-HT concentration-response curve. Data on vasomotor response were excluded from the study if segments did not respond to K^+^ stimulation, there were missing values in the cumulative concentration-response curves, data could not be fitted to a sigmoid curve with nonlinear regression analysis or if the segment could not be mounted in the organ bath due to technical difficulties. Animals from different exposure groups were analyzed on the same experimental day.

### Statistical analysis

All concentration-response curves were analyzed by non-linear regression analysis using GraphPad Prism version 7 (San Diego, CA, USA). The data were fitted to sigmoid curves with varying slopes according to the following equation: Y = Bottom + (Top – Bottom)/ (1 + 10^((Log EC50 – X) * Hill Slope)), where X is the logarithm of concentration and Y is the response. Y starts at Bottom and goes to Top with a sigmoid shape. The data points on each curve are expressed as mean ± SEM and n denotes the number of rats. Non-overlapping confidence intervals were considered to be statistically different. Values of E_max_ (top value) and Log EC_50_ values were compared.

Data on biomarkers measured in plasma was analyzed by one-way ANOVA with Dunnett’s post-tests. All statistical analyses were considered significant when *P* < 0.05.

## Abbreviations

5-HT: 5-hydroxytryptamine
ACh: Acetylcholine
ADMA: Asymmetric dimethylarginine
BH_2_: Dihydrobiopterin
BH_4_: Tetrahydrobiopterin
BW: Body weight
DHA: Dehydroascorbic acid
DLS: Dynamic light scattering
EDTA: Ethylenediamine tetraacetic acid
E_max_: Maximal effect value
eNOS: Endothelial nitric oxide synthase
FBS: Foetal bovine serum
HUVEC: Human umbilical vein endothelial cell
KPSS: Potassium physiological salt solution
LAD: Left anterior descending
L-arg: L-arginine
L-NAME: *N*^G^-nitro-L-arginine methyl ester, Log EC_50_
MDA: Malondialdehyde
NO: Nitric oxide
NTA: Nanoparticle tracking analysis
NTG: Nitroglycerin
PGF_2α_: Prostaglandin F_2α_
PSS: Physiological saline solution
